# Prevalence and radiological definitions of acetabular dysplasia after the age of 2 years: a systematic review

**DOI:** 10.1097/BPB.0000000000001113

**Published:** 2023-08-07

**Authors:** Suzanne de Vos-Jakobs, Fleur Boel, Wichor M. Bramer, Sita M.A. Bierma-Zeinstra, Rintje Agricola

**Affiliations:** a Department of Orthopaedics and Sports Medicine, Erasmus MC – Sophia Children’s Hospital, University Medical Centre Rotterdam; b Medical Library, University Medical Centre Rotterdam, Erasmus MC, Rotterdam; c Department of Orthopaedics, St. Anna Hospital, Geldrop, The Netherlands

**Keywords:** developmental dysplasia of the hip, epidemiology, osteoarthritis, radiography

## Abstract

Acetabular dysplasia is one of the most common causes of early hip osteoarthritis and hip replacement surgery. Recent literature suggests that acetabular dysplasia does not always originate at infancy, but can also develop later during childhood. This systematic review aims to appraise the literature on prevalence numbers of acetabular dysplasia in children after the age of 2 years. A systematic search was performed in several scientific databases. Publications were considered eligible for inclusion if they presented prevalence numbers on acetabular dysplasia in a general population of healthy children aged 2–18 years with description of the radiological examination. Quality assessment was done using the Newcastle-Ottawa score. Acetabular dysplasia was defined mild when: the center-edge angle of Wiberg (CEA-W) measured 15–20°, the CEA-W ranged between -1 to -2SD for age, or based on the acetabular index using thresholds from the Tönnis table. Severe dysplasia was defined by a CEA-W < 15°, <-2SD for age, or acetabular index according to Tönnis. Of the 1837 screened articles, four were included for review. Depending on radiological measurement, age and reference values used, prevalence numbers for mild acetabular dysplasia vary from 13.4 to 25.6% and for severe acetabular dysplasia from 2.2 to 10.9%. Limited literature is available on prevalence of acetabular dysplasia in children after the age of 2 years. Prevalence numbers suggest that acetabular dysplasia is not only a condition in infants but also highly prevalent later in childhood.

## Introduction

Developmental dysplasia of the hip (DDH) is the single most common musculoskeletal disorder in infants and young children. It occurs in 5–10% of live births throughout Western countries. DDH includes a broad spectrum of hip pathology from hip dislocation up to stable hips with acetabular dysplasia [1].

Treatment of DDH is based on preservation of the native hip joint and resolving acetabular dysplasia. Treating acetabular dysplasia is important in order to establish a wide load-bearing acetabular surface for evenly distributed weightbearing and therefore diminishing risk for osteoarthritis and total hip replacement in the long term [2].

However, despite all efforts in treating DDH during childhood, prevalence rates for acetabular dysplasia still remain high in adults (5–21%) [2–5]. This suggests that either current treatment is insufficient or that a large number of children who eventually develop acetabular dysplasia remain out of scope.

Interestingly, DDH is currently considered to originate in infants and babies. Screening programs, therefore, focus on diagnosis and treatment in the first months of life but do not take late-onset or developmental factors into account. However, other studies suggest that acetabular dysplasia can also develop later during growth [4,6] and might be influenced by environmental factors during childhood [7]. This might be one of the reasons that acetabular dysplasia often remains undiagnosed and untreated and therefore might (partially) explain the high prevalence of acetabular dysplasia in adults [8]

Another reason for the high prevalence numbers in adults can be due to radiological measurements used to quantify acetabular dysplasia. Most radiological measurements used for (residual) acetabular dysplasia are based on reference values for adults. Only Tönnis’ table for acetabular index provides data specific for age, gender and laterality during childhood up to 7 years of age [9]. If center-edge-angle of Wiberg (CEA-W) or the lateral center-edge-angle (LCEA) are used in children, reference values are based on adult values [6,10].

Therefore, this systematic review aims to appraise the literature on prevalence of acetabular dysplasia in the general population of children after the age of 2 years. Second, we aim to describe the radiological measurements used to diagnose acetabular dysplasia during childhood.

## Methods

The protocol for this systematic research was published in the PROSPERO database, reference number CRD42021282217.

### Data sources and study selection

The methods are described based on the Preferred reporting items for systematic reviews and meta-analyses (PRISMA) checklist [11] and the PRISMA-S extension to the PRISMA Statement for Reporting Literature Searches in Systematic Reviews [12]. An exhaustive search strategy was developed by an experienced information specialist (W.M.B.). The original search was developed in October 2021 in Embase.com, optimized for sensitivity, then translated to other databases and later updated in May 2022 following the method as described by Bramer *et al.* [13,14]. The search was carried out in the databases Embase.com (date of inception 1971), Medline ALL via Ovid (1946 to Daily Update), Web of Science Core Collection and the Cochrane Central Register of Controlled Trials via Wiley (date of inception 1992).

The search strategies for Embase and Medline used relevant thesaurus terms from Emtree and Medical Subject Headings (MeSH), respectively. In all databases, terms were searched in titles and abstracts of references. The search contained terms for (1) hip dysplasia or congenital hip dislocation and (2) either a combination of incidence or epidemiology in children or terms related to diagnostic delay or late presentation. Terms were combined with Boolean operators AND and OR and proximity operators were used to combined terms into phrases. The full search strategies of all databases are available in the supplementary materials (Appendix A, Supplemental digital content 1, http://links.lww.com/JPOB/A83). The searches in Embase and Web of Science were limited to exclude conference papers. In all databases, non-English articles, and animal-only articles were excluded from the search results. No study registries were searched, but Cochrane CENTRAL retrieves the contents of ClinicalTrials.gov and WHO’s International Clinical Trials Registry Platform. According to the methodology proposed by Bramer *et al.* [15–17] the following steps were taken: (1) the reference lists of retrieved non-included relevant review articles and of the included references, as well as articles citing these papers have been scanned for relevant references missed by the search; (2) the references were imported into EndNote and duplicates were removed; (3) two reviewers (S.d.V. and F.B.) independently screened titles and abstracts in EndNote. Any discrepancies in the verdict were resolved by discussion with a third reviewer (RA). Next, full texts were retrieved for (preliminary) included articles. Definite inclusion was done by reading full text of the remaining articles by two independent reviewers (S.d.V. and F.B.) and discrepancies were again resolved by a third reviewer (R.A.).

The inclusion and exclusion criteria are listed in Table 1.

**Table 1:** In- and exclusion criteria

Inclusion	Exclusion
Reported prevalence of acetabular dysplasia in children aged 2–18 years (or subgroup analysis within the age range)	Comorbidities compromising hip development (such as neuromuscular disorders or syndromal diseases)
Data from which prevalence numbers can be calculated (e.g. incidence and/or reference values)	Non-ambulatory children
General population (including children with and without acetabular dysplasia)	Solely hip dislocation
Diagnosis of acetabular dysplasia confirmed by any imaging modality (radiograph, DXA, ultrasound, CT, MRI)	

### Quality assessment

Quality assessment of the included articles was done, using the Newcastle-Ottawa scale (NOS) for cross-sectional studies [18]. This questionnaire was specified for the topic (Appendix B, Supplemental digital content 2, http://links.lww.com/JPOB/A84). Both reviewers (S.d.V. and F.B.) independently calculated a NOS score and discrepancies were again solved by the third reviewer (R.A.). The scores for 3 aspects of quality (selection, comparability and outcome) were separately used for the definite estimation of quality.

Studies that scored a total of 7 or 8 points were considered to have a low risk of bias; 6 points were considered to have a medium risk of bias; 5 points or less were considered to have a high risk of bias [18].

### Data extraction

Before reading the articles, a data extraction form was composed by the authors (Appendix C, Supplemental digital content 3, http://links.lww.com/JPOB/A85). This information was extracted by 2 reviewers (S.d.V. and F.B.) independently and discussed in order to achieve agreement. When provided, prevalence numbers for mild acetabular dysplasia (CEA-W or LCEA 15–20° or −1 to 2 SD; acetabular index determined by Tönnis) and severe acetabular dysplasia (CEA-W or LCEA < 15° or <2SD; acetabular index determined by Tönnis) will be reported separately for each study. If appropriate, prevalence data of studies will be pooled.

## Results

**Fig. 1:**
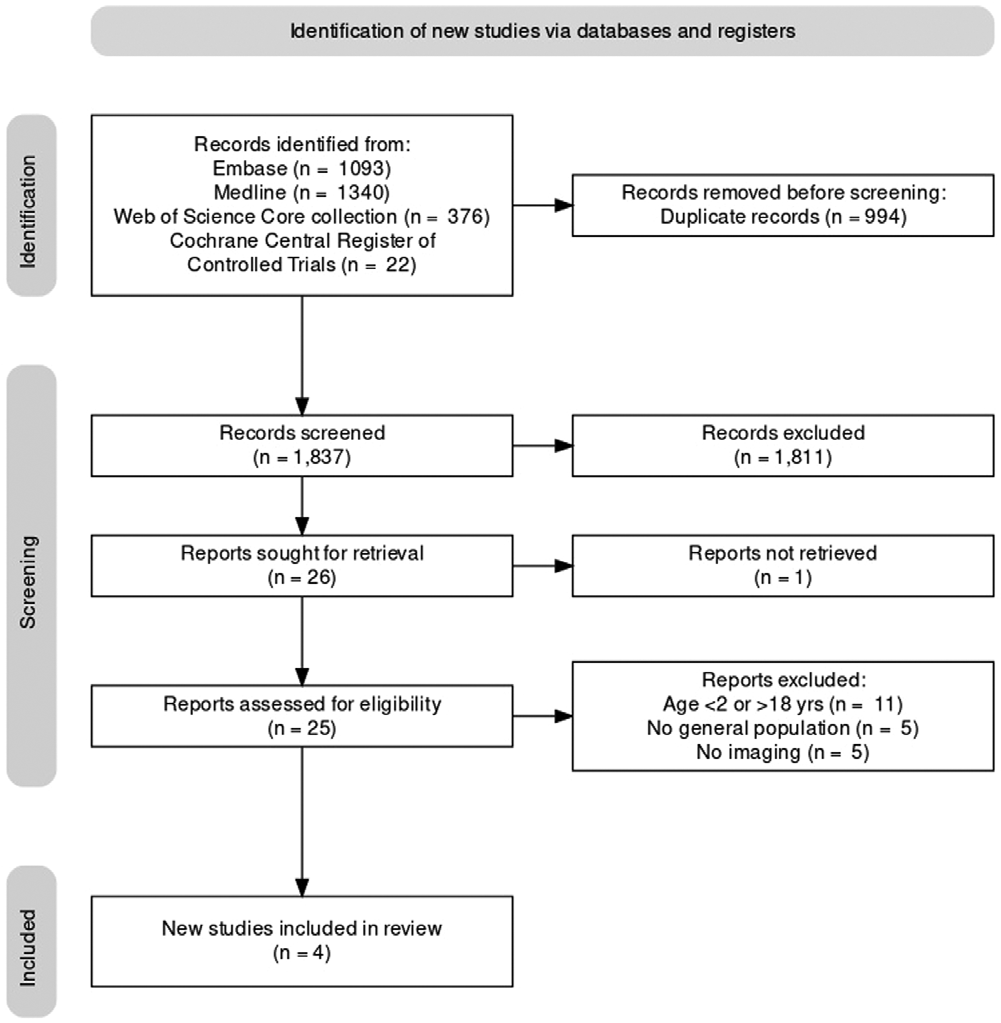
PRISMA flow diagram for article inclusion.

The systematic search resulted in a total of 1837 articles. Figure 1 shows the flow from the initial searches to the final inclusion of four articles.

### Quality assessment

The quality assessment for the four included articles is summarized in Table 2. Only the study by Chung *et al.* [7]. had an overall low risk of bias.

**Table 2:**
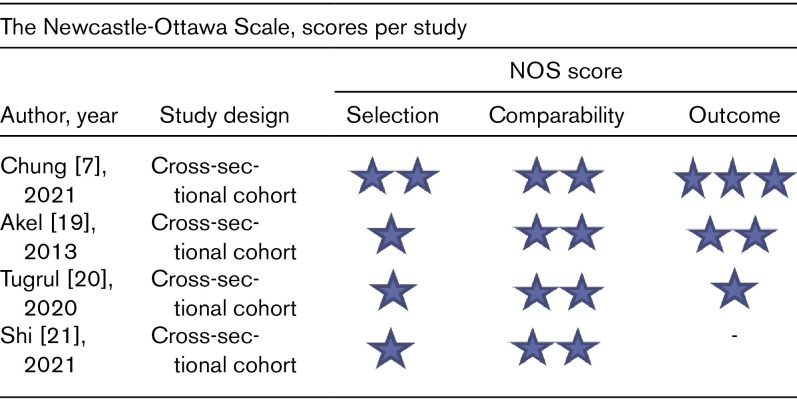
Quality assessment scores

### Prevalence

Pooling was not appropriate due to the heterogeneous character of the data. For one of the studies [22] the prevalence was calculated by ourselves, using the data and numbers from the reference values reported in the article.

In Table 3, the study characteristics and outcomes are summarized.

**Table 3:** Summary of study data

Summary of studies										
Author, year	Cohort characteristics				Imaging characteristics		Outcomes			
	Sample size (no. of hips)	Age (years)	Gender	Ethnicity	Imaging modality	Measurement	Cutoff value severe dysplasia	Prevalence severe dysplasia (%)	Cutoff value mild dysplasia	Prevalence mild dysplasia (%)
Chung, 2021 [7]	1026	9.86	♀ 49.8% ♂ 50.2%	European 79.5% Asian 7.7% African 11.2% Other 1.6%	HR-DXA	CEA-W	<15°	4.8	15–20°	25
Akel, 2013 [19]	4622	2–8	♀ 52.9% ♂ 47.1%	Turkish	Radiograph	Acetabular index	Defined by Tönnis	2.6–10.9	Defined by Tönnis	14.7–29.9
							>+2SD	2.9–4.8	+ 1 to 2 SD	14.9–21.6
Tugrul, 2020 [20]	3192	5–14	♀ 46.1% ♂ 53.9%	Turkish	Radiograph	CEA-W	<−2SD	2.21	−1 to 2 SD	14.06
Shi, 2021 [21]	1892	4–17	♀ 38.5% ♂ 61.5%	Multi-ethnical Chinese	Radiograph	CEA-W	<−2SD	2.2	−1 to 2SD	13.4

CEA-W, center-edge angle of Wiberg; HR-DXA, high-resolution dual-energy X-ray absorptiometry.

Chung *et al.* [7]. described the prevalence of acetabular dysplasia in a randomly selected cross-sectional subgroup of 9-year-olds from an ongoing prospective population-based cohort (Generation R). Besides CEA-W, acetabular depth-width ratio (ADR) was used to determine acetabular development. Since the cutoff values for ADR were chosen so, that the prevalence of acetabular dysplasia was similar to the prevalence measured with CEA-W, these measurements are not included in our systematic review.

Akel *et al.* [19]. Derived their population from a database of lower abdomen and pelvis radiographs for non-dysplasia-related causes. Age groups were defined per year. Cut-off values and prevalence vary by age group. More detailed information for the various age groups is attached in Appendix D, Supplemental digital content 4, http://links.lww.com/JPOB/A86.

Tugrul *et al.* [20]. used the same database as Akel *et al*. and defined similar age groups (ages 5–14 years, groups of 1 year). cutoff values for dysplasia were estimated by their own measurements (CEA-W −1 to −2 SD for mild dysplasia, CEA-W < −2SD for severe dysplasia). This resulted in reference values varying by age, gender and laterality (Appendix E, Supplemental digital content 5, http://links.lww.com/JPOB/A87).

Shi *et al.* [22]. designed their study to establish reference values for CEA-W for the Chinese population per age group. In order to do so, they used a database of radiographs ‘for routine examination or exclusion of pelvic trauma’. With the use of the 95% confidential interval, prevalence was calculated.

## Discussion

The overall prevalence of mild acetabular dysplasia in children aged 2 years and older is estimated between 13.4 and 25.6% and for severe acetabular dysplasia between 2.2 and 10.9% when measured by CEA-W or acetabular index. While acetabular dysplasia in otherwise healthy children is currently thought to develop during infancy and improve over time, these results show that prevalence remains high in later childhood. In the reviewed literature CEA-W and acetabular index are most widely used, but also ADR can be measured as indicator for acetabular dysplasia.

### Limited number of studies available on acetabular dysplasia during childhood

In the systematic search, only four articles met our inclusion criteria indicating that the number of studies on prevalence of acetabular dysplasia after the age of 2 years is limited. In contrast, large numbers of studies were published on prevalence of DDH during infancy [21,23] or prevalence of late-diagnosed hip dislocation [24,25]. Also, when numbers on DDH after the age of 2 years were presented, this was not in the general population, but in more biased populations such as hospitalized patients. For that reason further (longitudinal) research in the general population is essential to acquire more information on the development of acetabular dysplasia during growth.

### Patient selection and representation

Of the four included studies, only one study (Chung *et al*.) was an unadulterated general (multi-ethnic) population study where high-resolution DXA’s were derived for research purpose only and not for treatment or diagnostic purposes. All other studies used radiographs that were made for other purposes but considered it a sample of the general population as no hip complaints were reported. Akel *et al*. and Tugrul *et al*. both used radiographs from the same database derived for ‘non-dysplasia related causes’, but no information was provided on the indication for radiographs. Healthy, non-complaining children will probably not routinely have this radiograph obtained and therefore a potential selection bias cannot be ruled out. Similarly, Shi *et al*. used radiographs taken for ‘routine examination or exclusion of pelvic trauma’, probably in an emergency setting, but this information is not provided.

Outcomes of the study of Shi *et al*. might be less representative for populations outside China. Far more male participants than female participants were included, and it is known that acetabular dysplasia is more common in females than in males [1]. Therefore, prevalence numbers for the general population might be underestimated. On the other hand, prevalence of acetabular dysplasia is known to be higher in Asian populations compared to Caucasian populations [26]. Altogether, we conclude that the prevalence numbers from this study are less representative of non-Chinese population than the prevalence numbers of the other reviewed studies.

### Prevalence numbers and reference values

Both the study of Tugrul *et al*. and Shi *et al*. used their own calculated cutoff values to estimate the prevalence numbers of acetabular dysplasia based on the 95% confidential interval. As a result of this method, one can anticipate that prevalence numbers will be close to 13.6% for mild acetabular dysplasia (<−1 SD) and 2.2% for severe acetabular dysplasia (<−2 SD). In normally distributed data these percentages represent ±1 SD and ±2 SD, respectively. For that reason, the prevalence number derived from these studies are less informative than from the study of Chung *et al*. and Akel *et al*.

Also, for the estimation of cutoff values, this might not be the optimal approach. With this method, the assumption is made that 13.6% of the population has mild acetabular dysplasia and 2.2% has severe dysplasia, but this might be incorrect, given prevalence numbers of acetabular dysplasia in adults [3,4,8]. As the spectrum of DDH is more common in females than in males [1], this calculation method results in gender-specific normal values (−1 SD and −2 SD are at different values), ultimately leading to either overestimation of acetabular dysplasia in males or underestimation of acetabular dysplasia in females. A more reliable method to establish normal values would be using a certain outcome in time such as developing hip symptoms in young adulthood or osteoarthritis later in life. At skeletal maturity hip joints of both males and females equally require a wide load-bearing acetabular surface for diminishing risk of hip complaints in the long term [2]. Preferably longitudinal studies should be performed, where these pathological outcomes can be correlated to acetabular development and threshold values in children.

Only Akel *et al*. present prevalence numbers based on previously verified cutoff values for the specific age groups (Tönnis’ table for acetabular index). Still, the prevalence of acetabular dysplasia in this study is equally as high as in the other reviewed studies.

### Definition of radiological measurements

The studies of Chung *et al*., Tugrul *et al*. and Shi *et al*. refer to their measurements as ‘center-edge angle of Wiberg’. Chung *et al*. and Tugrul *et al*. specify in their text how the measurement is performed, while Shi *et al*. don’t provide details on the measurement. Based on the recent consensus on measurement of the center-edge angle [27], we conclude that both Chung *et al*. and Tugrul *et al*. have actually reported the lateral center-edge angle (LCEA) instead of the CEA-W. LCEA refers to the most lateral point of the acetabulum and CEA-W refers to the most lateral point of the acetabular source, so these values aren’t always equal. Especially in dysplastic hips, CEA-W is often lower than LCEA. These studies use reference values for (the lower) CEA-W and compare them with a measured (mostly higher) LCEA, this may lead to an underestimation of the prevalence of acetabular dysplasia in both studies.

### Conclusion

Prevalence of mild acetabular dysplasia in children over 2 years of age is 13.4–25.6%, and for severe dysplasia prevalence is 2.2–10.9%. Very limited data is available, but based on the reviewed data, prevalence of acetabular dysplasia varies strongly by age, method of measuring, and estimated cutoff values.

Either way, acetabular dysplasia not only seems to be a condition in infants but is also of great importance later in childhood. For this reason, health care practitioners should be more suspicious for acetabular dysplasia, also when DDH is ruled out during infancy. Future longitudinal studies in general, multi-ethnic populations are essential for evaluation of acetabular development, its determinants and prognostic implications.

## Acknowledgements

### Conflicts of interest

There are no conflicts of interest.

## Supplementary Material










