# Supplementary material for: Prevalence and radiological definitions of acetabular dysplasia after the age of 2 years: a systematic review
Source: J Pediatr Orthop B. 2023 Aug 7;33(4):334–9. doi: 10.1097/BPB.0000000000001113 (PMC11132094; doi:10.1097/BPB.0000000000001113)
Supplement: Supplementary file 1 [file jpob-33-334-s001.pdf]

Appendix A – Complete search strategy

Embase.com

('hip dysplasia'/exp OR 'congenital hip dislocation'/de OR ('congenital joint dislocation'/de AND (hip/de OR 'hip dislocation')) OR 'hip dislocation'/exp/dm_cn OR 'acetabular dysplasia'/de OR (((hip OR hips OR Acetabul*) NEAR/6 (dysplas*)) OR ((hip OR hips OR Acetabul*) NEAR/6 (development* OR congenital*) NEAR/6 (dislocat*))):ab,ti) AND (((incidence/exp OR epidemiology/de OR 'hip dysplasia'/exp/dm_ep OR 'hip dislocation'/exp/dm_ep OR prevalence/de OR screening/exp OR morbidity/de OR 'geographic distribution'/de OR (inciden* OR epidemiolog* OR prevalen* OR screening OR frequen* OR morbidit* OR (geograph* NEAR/3 distribut*)):Ab,ti) AND ('preschool child'/exp OR child/de OR adolescent/de OR childhood/de OR adolescence/de OR (preschool* OR pre-school* OR (child* NEAR/6 (2 OR 3 OR 4 OR 5) NEXT/1 (year*)) OR (child* NEAR/6 age* NEXT/1 (2 OR 3 OR 4 OR 5 OR 6 OR 7 OR 8 OR 9 OR 10 OR 11 OR 12 OR 13 OR 14 OR 15 OR 16)) OR (age* NEXT/1 (2 OR 3 OR 4 OR 5 OR 6 OR 7 OR 8 OR 9 OR 10 OR 11 OR 12 OR 13 OR 14 OR 15 OR 16) NEXT/1 year*) OR (child* NEAR/6 (24 OR 36 OR 48 OR 60 OR 72 OR 84 OR 96 OR 108 OR 120 OR 132 OR 144 OR 156 OR 168 OR 180 OR 192) NEXT/1 month*)):Ab,ti)) OR ('missed diagnosis'/exp OR 'delayed diagnosis'/de OR 'diagnostic delay'/de OR 'onset age'/de OR 'diagnostic error'/exp OR (((late* OR delay* OR missed OR Enhanc* OR error*) NEAR/3 (presentation* OR diagnos* OR detect* OR onset*)) OR (false NEAR/3 negative*)):Ab,ti)) NOT [conference abstract]/lim NOT ([animals]/lim NOT [humans]/lim) AND [english]/lim

Medline ALL Ovid

(Hip Dislocation/ OR Hip Dislocation, Congenital / OR Developmental Dysplasia of the Hip/ OR (Joint Dislocations AND (Hip / OR Hip Dislocation/)) OR (((hip OR hips OR Acetabul*) ADJ6 (dysplas*)) OR ((hip OR hips OR Acetabul*) ADJ6 (development* OR congenital*) ADJ6 (dislocat*))).ab,ti.) AND (((Incidence/ or Epidemiology/ or Prevalence/ or Mass Screening/ or Morbidity/ OR Hip Dislocation/ep OR (inciden* OR epidemiolog* OR prevalen* OR screening OR frequen* OR morbidit* OR (geograph* ADJ3 distribut*)).ab,ti.) AND (Child, Preschool/ OR Child/ OR Adolescent/ OR (preschool* OR pre-school* OR (child* ADJ6 (2 OR 3 OR 4 OR 5) ADJ (year*)) OR (child* ADJ6 age* ADJ (2 OR 3 OR 4 OR 5 OR 6 OR 7 OR 8 OR 9 OR 10 OR 11 OR 12 OR 13 OR 14 OR 15 OR 16)) OR (age* ADJ (2 OR 3 OR 4 OR 5 OR 6 OR 7 OR 8 OR 9 OR 10 OR 11 OR 12 OR 13 OR 14 OR 15 OR 16) ADJ year*) OR (child* ADJ6 (24 OR 36 OR 48 OR 60 OR 72 OR 84 OR 96 OR 108 OR 120 OR 132 OR 144 OR 156 OR 168 OR 180 OR 192) ADJ month*)).ab,ti.)) OR (Missed Diagnosis/ or Delayed Diagnosis/ OR Age of Onset/ OR exp Diagnostic Errors/ OR (((late* OR delay* OR missed OR Enhanc* OR error*) ADJ3 (presentation* OR diagnos* OR detect* OR onset*)) OR (false ADJ3 negative*)).ab,ti.)) NOT (conference abstract) NOT (exp animals/ NOT humans/) AND english.la.

Web of Science Core Collection*

*Science Citation Index Expanded (1975-present) ; Social Sciences Citation Index (1975-present) ; Arts & Humanities Citation Index (1975-present) ; Conference Proceedings Citation Index- Science (1990-present) ; Conference Proceedings Citation Index- Social Science & Humanities (1990-present) ; Emerging Sources Citation Index (2015-present)

TS=(((((hip OR hips OR Acetabul*) NEAR/5 (dysplas*)) OR ((hip OR hips OR Acetabul*) NEAR/5 (development* OR congenital*) NEAR/5 (dislocat*)))) AND ((((inciden* OR epidemiolog* OR prevalen* OR screening OR frequen* OR morbidit* OR (geograph* NEAR/2 distribut*))) AND ((preschool* OR pre-school* OR (child* NEAR/5 (2 OR 3 OR 4 OR 5) NEAR/1 (year*)) OR (child* NEAR/5 age* NEAR/1 (2 OR 3 OR 4 OR 5 OR 6 OR 7 OR 8 OR 9 OR 10 OR 11 OR 12 OR 13 OR 14 OR 15 OR 16)) OR (age* NEAR/1 (2 OR 3 OR 4 OR 5 OR 6 OR 7 OR 8 OR 9 OR 10 OR 11 OR 12 OR 13 OR 14 OR 15 OR 16) NEAR/1 year*) OR (child* NEAR/5 (24 OR 36 OR 48 OR 60 OR 72 OR 84 OR 96 OR 108 OR 120 OR 132 OR 144 OR 156 OR 168 OR 180 OR 192) NEAR/1 month*)))) OR ((((late* OR delay* OR missed OR Enhanc* OR error*) NEAR/2 (presentation* OR diagnos* OR detect* OR onset*)) OR (false NEAR/2 negative*)))))

Cochrane Central Register of Controlled Trials

((((hip OR hips OR Acetabul*) NEAR/6 (dysplas*)) OR ((hip OR hips OR Acetabul*) NEAR/6 (development* OR congenital*) NEAR/6 (dislocat*))):ab,ti) AND ((((inciden* OR epidemiolog* OR prevalen* OR screening OR frequen* OR morbidit* OR (geograph* NEAR/3 distribut*)):Ab,ti) AND ((preschool* OR pre-school* OR (child* NEAR/6 (2 OR 3 OR 4 OR 5) NEXT/1 (year*)) OR (child* NEAR/6 age* NEXT/1 (2 OR 3 OR 4 OR 5 OR 6 OR 7 OR 8 OR 9 OR 10 OR 11 OR 12 OR 13 OR 14 OR 15 OR 16)) OR (age* NEXT/1 (2 OR 3 OR 4 OR 5 OR 6 OR 7 OR 8 OR 9 OR 10 OR 11 OR 12 OR 13 OR 14 OR 15 OR 16) NEXT/1 year*) OR (child* NEAR/6 (24 OR 36 OR 48 OR 60 OR 72 OR 84 OR 96 OR 108 OR 120 OR 132 OR 144 OR 156 OR 168 OR 180 OR

192) NEXT/1 month*)):Ab,ti)) OR ((((late* OR delay* OR missed OR Enhanc* OR error*) NEAR/3 (presentation* OR diagnos* OR detect* OR onset*)) OR (false NEAR/3 negative*)):Ab,ti))
