# Supplementary material for: Prevalence and radiological definitions of acetabular dysplasia after the age of 2 years: a systematic review
Source: J Pediatr Orthop B. 2023 Aug 7;33(4):334–9. doi: 10.1097/BPB.0000000000001113 (PMC11132094; doi:10.1097/BPB.0000000000001113)
Supplement: Supplementary file 2 [file jpob-33-334-s002.pdf]

Appendix B – Newcastle-Ottawa Scale for Quality Assessment of Cross-Sectional Studies

Selection (maximum 3)

1. Representativeness of the sample
    a. Truly representative of the average in the target population (random sample or whole population) *
    b. Somewhat representative of the average in the target population *
    c. Selected group
    d. No description of the sampling strategy
2. Sample size
    a. Justified and satisfactory (>1000 total)*
    b. Not justified
3. Non-included subjects
    a. Comparability between subjects and non-subjects characteristics is established*
    b. The response rate is unsatisfactory
    c. No description of the response rate

Comparability (maximum 2)

1. The subjects in different outcome groups are comparable, based on the study design or analysis. Confounding factors are controlled
    a. Study controls for age and gender (or analysis separated by gender)*
    b. Study controls for any additional factor *

Outcome (maximum 3)

1. Assessment of the outcome (hip morphology measurement)
    a. Blind*
    b. Description measurement*
    c. No description
2. Statistical test
    a. The statistical test used to analyze the data was clearly described and appropriate, and the measurement of the association was presented, including confidence intervals and the probability level (p value)*
    b. The statistical test is not appropriate, not described or incomplete
