# Supplementary material for: Prevalence and radiological definitions of acetabular dysplasia after the age of 2 years: a systematic review
Source: J Pediatr Orthop B. 2023 Aug 7;33(4):334–9. doi: 10.1097/BPB.0000000000001113 (PMC11132094; doi:10.1097/BPB.0000000000001113)
Supplement: Supplementary file 3 [file jpob-33-334-s003.pdf]

Appendix C – Data extraction form

	Study design	Population characteristics					Imaging		
		Number		Age group	Cohort / sample	Etnicity	Modality	Measurement(s)	Observers
		Hips	Patients						No. and type
Akel									
Shi									
Tugrul									
Chung									

	Prevalence											
	Reported						Calculated					
	Mild (n)	Mild (%)	Severe (n)	Severe (%)	Mean	SD	Cut-off Mild	Cut-off severe	Mild (n)	Mild (%)	Severe (n)	Severe (%)
Akel												
Shi												
Tugrul												
Chung												
