# Supplementary material for: Prevalence and radiological definitions of acetabular dysplasia after the age of 2 years: a systematic review
Source: J Pediatr Orthop B. 2023 Aug 7;33(4):334–9. doi: 10.1097/BPB.0000000000001113 (PMC11132094; doi:10.1097/BPB.0000000000001113)
Supplement: Supplementary file 4 [file jpob-33-334-s004.pdf]

# Appendix D – Specifications from Akel et al.

Tables copied from original article.(21)

| Age (yrs.) | Male | | | | | | Female | | | | | |
|---|---|---|---|---|---|---|---|---|---|---|---|---|
| | Right | | | Left | | | Right | | | Left | | |
| | N | Mild dys. | Severe dys. | N | Mild dys. | Severe dys. | N | Mild dys. | Severe dys. | N | Mild dys. | Severe dys. |
| [illegible] | [illegible] | [illegible] | [illegible] | [illegible] | [illegible] | [illegible] | [illegible] | [illegible] | [illegible] | [illegible] | [illegible] | [illegible] |
| 2 | [illegible] | [illegible] | [illegible] | [illegible] | [illegible] | [illegible] | [illegible] | [illegible] | [illegible] | [illegible] | [illegible] | [illegible] |
| 3 | <21 | 21-24 | >24 | <21 | 21-25 | >25 | <23 | 23-27 | >27 | <23 | 23-26 | >26 |
| 4 | <20 | 20-24 | >24 | <20 | 20-24 | >24 | <21 | 21-25 | >25 | <21 | 21-25 | >25 |
| 5 | <19 | 19-22 | >22 | <19 | 19-23 | >23 | <20 | 20-23 | >23 | <20 | 20-24 | >24 |
| 6 | <18 | 18-21 | >21 | <18 | 18-22 | >22 | <20 | 20-24 | >24 | <20 | 20-23 | >23 |
| 7 | <18 | 18-21 | >21 | <18 | 18-21 | >21 | <18 | 18-22 | >22 | <18 | 18-22 | >22 |
| 8 | <17 | 17-21 | >21 | <17 | 17-21 | >21 | <18 | 18-23 | >23 | <19 | 19-23 | >23 |

N: normal, dys.: dysplasia

Cut-off values from own calculation

| Age (yrs.) | Study values | | | | Tönnis | | | | p | p (Mild) | p (Severe) |
|---|---|---|---|---|---|---|---|---|---|---|---|
| | Mild dysplasia rate | | Severe dysplasia rate | | Mild dysplasia rate | | Severe dysplasia rate | | | | |
| | N | Rate (%) | N | Rate (%) | N | Rate (%) | N | Rate (%) | | | |
| 1 | [illegible] | [illegible] | [illegible] | [illegible] | [illegible] | [illegible] | [illegible] | [illegible] | [illegible] | [illegible] | [illegible] |
| 2 | [illegible] | [illegible] | [illegible] | [illegible] | [illegible] | [illegible] | [illegible] | [illegible] | [illegible] | [illegible] | [illegible] |
| 3 | 65 | 17 | 18 | 4.7 | 56 | 14.7 | 20 | 5.2 | 0.336 | 0.108 | 0.687 |
| 4 | 46 | 14.9 | 16 | 5.2 | 92 | 29.9 | 21 | 6.8 | <0.001 | <0.001 | 0.125 |
| 5 | 60 | 19.1 | 13 | 4.2 | 77 | 24.6 | 8 | 2.6 | 0.250 | 0.035 | 0.125 |
| 6 | 66 | 21.6 | 12 | 3.9 | 81 | 26.4 | 14 | 4.6 | <0.001 | <0.001 | 0.754 |
| 7 | 52 | 16.8 | 13 | 4.2 | 66 | 21.3 | 8 | 2.6 | 0.414 | 0.007 | 0.180 |
| 8 | 52 | 18.5 | 8 | 2.8 | | | | | | | |
| Total | 493 | 17.8 | 118 | 4.3 | 604 | 24.3 | 159 | 6.4 | <0.001 | <0.001 | <0.001 |

N: number

Acetabular dysplasia rates
