# Supplementary material for: Prevalence and radiological definitions of acetabular dysplasia after the age of 2 years: a systematic review
Source: J Pediatr Orthop B. 2023 Aug 7;33(4):334–9. doi: 10.1097/BPB.0000000000001113 (PMC11132094; doi:10.1097/BPB.0000000000001113)
Supplement: Supplementary file 5 [file jpob-33-334-s005.pdf]

Appendix E – Specifications Tugrul et al.

Table copied from original article.(22)

	Male						Female					
	Right			Left			Right			Left		
Age	N	Mild	Severe	N	Mild	Severe	N	Mild	Severe	N	Mild	Severe
5	>20	15-20	<15	>20	15-20	<15	>19	14-19	<14	>19	14-19	<14
6	>20	15-20	<15	>20	15-20	<15	>19	14-19	<14	>20	15-20	<15
7	>20	15-20	<15	>21	16-21	<16	>20	15-20	<15	>21	16-21	<16
8	>20	15-20	<15	>21	16-21	<16	>21	16-21	<16	>21	16-21	<16
9	>21	16-21	<16	>22	17-22	<17	>21	16-21	<16	>22	17-22	<17
10	>21	16-21	<16	>22	17-22	<17	>21	16-21	<16	>22	17-22	<17
11	>22	17-22	<17	>22	17-22	<17	>21	16-21	<16	>23	18-23	<18
12	>23	18-23	<18	>23	18-23	<18	>22	17-22	<17	>23	18-23	<18
13	>23	18-23	<18	>23	18-23	<18	>22	17-22	<17	>23	18-23	<18
14	>23	18-23	<18	>23	18-23	<18	>22	17-22	<17	>23	18-23	<18

N: normal

Cut-off values for acetabular dysplasia from Tugrul et al.(22)
